# Green Synthesis of Silver Nanoparticles Using the Plant Extract of *Acer oblongifolium* and Study of Its Antibacterial and Antiproliferative Activity via Mathematical Approaches

**DOI:** 10.3390/molecules27134226

**Published:** 2022-06-30

**Authors:** Muhammad Naveed, Bakhtawar Bukhari, Tariq Aziz, Sumera Zaib, Muhammad Adil Mansoor, Ayaz Ali Khan, Muhammad Shahzad, Anas S. Dablool, Mashael W. Alruways, Abdulraheem Ali Almalki, Abdulhakeem S. Alamri, Majid Alhomrani

**Affiliations:** 1 Department of Biotechnology, Faculty of Life Sciences, University of Central Punjab, Lahore 54590, Pakistan; bakhtawarb120@gmail.com; 2 Pak-Austria Fachhochschule, Institute of Applied Sciences and Technology, Mang, Haripur 22621, Pakistan; 3 Department of Biochemistry, Faculty of Life Sciences, University of Central Punjab, Lahore 54590, Pakistan; sumera.zaib@ucp.edu.pk; 4 Department of Chemistry, School of Natural Sciences (SNS), National University of Sciences and Technology (NUST), H-12, Islamabad 44000, Pakistan; adil.mansoor@sns.nust.edu.pk; 5 Department of Biotechnology, University of Malakand, Chakdara 18800, Pakistan; ayazkhan@uom.edu.pk; 6 Institute of Basic Medical Sciences, Khyber Medical University, Peshawar 25120, Pakistan; shahzad.ibms@kmu.edu.pk; 7 Department of Public Health, Health Sciences College Al-Leith, Umm Al-Qura University, Makkah al-Mukarramah 24382, Saudi Arabia; asdablool@uqu.edu.sa; 8 Department of Clinical Laboratory Sciences, College of Applied Medical Sciences, Shaqra University, Shaqra 15273, Saudi Arabia; m.alruways@su.edu.sa; 9 Department of Clinical Laboratory Sciences, The Faculty of Applied Medical Sciences, Taif University, P.O. Box 11099, Taif 21944, Saudi Arabia; almalki@tu.edu.sa (A.A.A.); a.alamri@tu.edu.sa (A.S.A.); m.alhomrani@tu.edu.sa (M.A.)

**Keywords:** green synthesis, silver nanoparticles, antibacterial activity, antiproliferative activity

## Abstract

In this study, the antibacterial and antifungal properties of silver nanoparticles synthesized with the aqueous plant extract of *Acer oblongifolium* leaves were defined using a simplistic, environmentally friendly, reliable, and cost-effective method. The aqueous plant extract of *Acer oblongifolium*, which served as a capping and reducing agent, was used to biosynthesize silver nanoparticles. UV visible spectroscopy, X-ray diffraction (XRD), Fourier Transform Infrared Spectroscopy (FTIR), and scanning electron microscopy were used to analyze the biosynthesized *Acer oblongifolium* silver nanoparticles (AgNPs). Gram-positive bacteria (*Bacillus paramycoides* and *Bacillus cereus*) and Gram-negative bacteria (*E. coli*) were used to test the AgNPs’ antibacterial activity. The presence of different functional groups was determined by FTIR. The AgNPs were rod-like in shape. The nanoparticles were more toxic against *Escherichia* *coli* than both *Bacillus cereus* and *Bacillus paramycoides*. The AgNPs had IC_50_ values of 6.22 and 9.43 and mg/mL on HeLa and MCF-7, respectively, proving their comparatively strong potency against MCF-7. This confirmed that silver nanoparticles had strong antibacterial activity and antiproliferative ability against MCF-7 and HeLa cell lines. The mathematical modeling revealed that the pure nanoparticle had a high heat-absorbing capacity compared to the mixed nanoparticle. This research demonstrated that the biosynthesized *Acer oblongifolium* AgNPs could be used as an antioxidant, antibacterial, and anticancer agent in the future.

## 1. Introduction

Nanotechnology has burgeoned as one of the most exciting and vast fields of study, with great opportunities [1]. Owing to the idiosyncratic properties of nanoparticles, including the high ratio of surface area to volume and magnetic, mechanical, optical, and chemical properties, they have remarkable prospects in emerging fields such as food, biomedicine, agriculture, and genetics [2]. Noble metal NPs, such as copper, silver, platinum, gold, zinc, magnesium, and titanium, have received a lot of recognition for their multifunctional theragnostic abilities in biomedical applications [3].

Nanoparticle synthesis has been achieved using chemical and physical techniques; however, these methods are not beneficial to the environment. As nanoparticles include plant extracts, animal proteins, agro-waste, pigments, bacteria, fungi, and small viruses, which are difficult to handle with conventional methods, green synthesis of nanoparticles is recommended [4]. Plants also include bioactive secondary metabolites such as aldehydes, ketones, terpenoids, polyphenols, tannins, polysaccharides, flavonoids, proteins, amines, and alkaloids, which serve as stabilizing and capping agents, and can reduce metal ions into metal nanoparticles, synthesizing required NPs with previously reported beneficial properties [5]. 

The actual situation of the pandemic fetched silver nanoparticles to the attention of researchers with special characteristics concerning antibacterial and antiviral protection [6]. Silver nanoparticles are among the most widely studied nanoparticles because they have flourished their wide applicability in the fields of biomedical applications, catalysis, electronics, and antimicrobial applications [7]. The last decade has seen a rise in research in the field of green synthesis of silver nanoparticles owing to environmental concerns [7]. To broaden the knowledge in the field of green synthesis of nanoparticles that have catalytic activity, biosynthesis of silver nanoparticles using spent coffee grounds for catalytic degradation of an organic contaminant has also been reported [8].

The properties of nanoparticles are exceptionally sensitive to their morphology, which increases the capability for their applications but at the same time makes their precise and reproduceable synthesis imperative [7]. Higher concentrations of silver are toxic; the literature shows that lower concentrations of AgNO_3_ have better intrinsic therapeutic prospects, catalytic activity, chemical stability, and biocompatibility [9]. Compared to bulk metals and their salts, AgNPs have been reported to have anticancer and antimicrobial activity [10]. Among the best attributes of silver nanoparticles is the gradual and controlled release of silver. Moreover, Padnya et al. [11], in their review, discussed the attractiveness of silver nanoparticles as an alternative to costly gold nanoparticles. AgNPs, apart from being used for medical purposes, are being exploited for their redox stability in biosensors with the assistance of cyclophanes [11].

*Acer oblongifolium*, also known as Himalayan maple, Kashmir maple, or evergreen maple, belongs to an Asian maple species of the soapberry family [12]. It is a medium-sized, perennial to semi-deciduous tree that grows to around 15–22 m (49–72 feet) in height [13]. This maple is one of the few that remains green in winter. *Acer oblongifolium* is found in eastern, central, and southeastern Asia, from northeast India and Tibet to Japan, including northern Indochina and southern China [14]. 

Researchers have explored the medical importance of this plant species extensively, but it has yet to be explored for its possible use in stabilizing and reducing the effects of green chemistry [15]. It has some medicinal properties with anti-tumor and antimicrobial activity, as well as a higher polyphenol and flavonoid content, which plays an important part in reducing metallic salt in stable nanoparticles. 

Antibiotic resistance among microbes, on the other hand, is becoming a global concern because of its widespread use. Noble metals (including Au, Ti, Pt, Pd, and Ag) have long been known to be effective antimicrobials against bacteria such as *Staphylococcus aureus*, *Klebsiella pneumoniae*, *Pseudomonas aeruginosa*, and *Salmonella typhimurium*, as well as fungi such as *Rhizoctonia solani, Aspergillus niger*, and *Candida* [16]. 

Additionally, AgNPs have not been fully explored in terms of their utilization against pathogens. These include *Escherichia coli*, a Gram-negative bacterium that causes cholecystitis, bacteremia, cholangitis, urinary tract infection, and traveler’s diarrhea [17]. *Bacillus cereus* is a Gram-positive foodborne pathogen that can produce toxins and cause emetic (vomiting) and diarrheal syndromes. *Bacillus cereus*, on the contrary, plays a part in fire blight, a contagious plant disease. *Bacillus paramycoides* is a Gram-positive bacterium that causes necrotic lesions in channel catfish [18]. Several studies, including the recent work of Platania et al., 2022 [19], have focused on the potential of AgNPs against many of these pathogens. In one such study, three colloidal suspensions of silver nanoparticles with different ionic ratios were synthesized and characterized for antibacterial activity. This study provided some of the first evidence that AgNPs’ bactericidal activity increases along with an increase in the concentration of silver nanoparticles [19]. 

In a similar study, the green synthesis of silver nanoparticles was orchestrated with the aim of increasing crop production, growth, and resistance to disease. The synthesized nanoparticles were analyzed for their antifungal and antibacterial properties against several plant pathogens and strengthened the foundation for the synthesis of silver nanoparticles [20]. Following this, the current study reports an unprecedented, one-step, cost-effective method for AgNPs biosynthesis at room temperature from *Acer oblongifolium* plant leave extract. Antimicrobial activity against pathogenic bacteria was also investigated; cytotoxicity assay, as well as physicochemical parameters of AgNPs, were assessed using mathematical approaches [21]. In this study, AgNPs were synthesized using the plant extract *Acer Oblongifolium,* and antibacterial activity against pathogenic bacteria with antiproliferative activity against MCF-7 and HeLa cell lines was shown.

The current study is different from previous studies in many respects. First, in this study, the *Acer oblongifolium* plant was used for silver nanoparticle synthesis for the first time. This plant has no use in any kind of nanoparticle formation in previously reported studies. Furthermore, the physiochemical properties (e.g., the size or shape) of synthesized nanoparticles are proximal to standards and have strong biological potency for exploration of their nanodelivery aspect. Moreover, the velocity and temperature profiles of pure nanoparticles and mixed nanoparticles (AgNPs mixed with water) are given for the first time in the current study of a green synthesis approach. Additionally, the IC50 values in the antiproliferative activity performed in the current study were far lower than in some other recent studies, revealing that the nanoparticles synthesized in the current study have more cytotoxic potential than previously synthesized nanoparticles [22,23,24,25].

## 2. Materials and Methods

### 2.1. Sample Collections 

*Acer oblongifolium* plant cultivated in the botanical garden of University of Punjab, Lahore, was collected in November 2021, and fresh leaves were separated and further processed for preparation of leaf extract.

### 2.2. Laboratory Preparation of Extracts from Plants

*Acer oblongifolium* fresh leaves were cleaned by washing under tap water and then rinsed with deionized water. The leaves were then shadow dried for 2–3 days and ground into powder using an electric blender. Leaf powder of 4 g was suspended in distilled water (200 mL) and kept in a water bath at a temperature of 70–80 °C for 30 min in a beaker. The remaining extracts were filtered in a conical flask using Whatman Grade 1 Filter Paper, cooled down, and refrigerated at 4 °C for further use in the synthesis of AgNPs according to the method described by Krithiga et al. and Pirtarighat et al. [26,27].

AgNO_3_ (25 mM, 1 M) solution was made by mixing AgNO_3_ flakes in deionized water. This solution was used for synthesizing AgNPs with the aim of testing their antibacterial activity.

### 2.3. Green Synthesis of AgNPs from the Extract

The solution of AgNO_3_ (25 mM, 1 M) was made by mixing AgNO_3_ flakes in deionized water, followed by AgNO_3_ and leaf extracts to the ratio of 1:10 *v*/*v*, respectively, to produce a volume of 50 mL in a reagent bottle wrapped with aluminum foil. The mixtures were incubated at room temperature until the yellow color of the solution turned dark brown. The samples were then centrifuged at 5000 revolutions per minute for 20 min, and the supernatant was discarded. Deionized water (5 mL) was added to the precipitate and centrifuged again with the same specifications. The process was repeated. The final precipitate was then placed in a hot air oven for 30 min at 60 °C, and the dried form was utilized in further testing.

### 2.4. AgNPs Characterization

#### 2.4.1. UV Visible Spectroscopy

The most important and simple technique for confirming the formation of nanoparticles is ultraviolet visible (UV Vis) spectrophotometry. AgNP formation was verified using a UV visible spectrophotometer, which monitored the band (300–700 nm) of surface plasmon resonance. 

#### 2.4.2. X-ray Diffraction (XRD) Analysis

The crystalline nature of the biosynthesized AgNPs was analyzed using X-ray diffraction (XRD). A powdered sample was utilized, and in the scanning mode—operated at 30 mA current with 40 kV voltage and Cu/Kα radiation with 20°–70° in 2*θ* angles—the diffraction pattern was recorded. The average crystalline size of AgNPs was calculated using the Debye–Scherrer equation. The equation is as follows:

*D = kλβcosθ* where *k* = shape factor (0.94).
*λ* = X-ray wavelength (*λ* = 1.5418 Å);
*β* = full width at half maximum (FWHM) in radians.
and *θ* = Bragg’s angle.

#### 2.4.3. Fourier Transform InfraRed (FTIR) Spectrum

The Fourier Transform InfraRed Spectrophotometer (FTIR, Bruker, Billerica, MA, USA) was used to determine the functional groups responsible for the synthesis of silver nanoparticles and record the FTIR spectrum. These functional groups may aid in the capping, reduction, and stabilization of silver nanoparticles. FTIR was performed in the spectral array from 400–4000 cm^−1^. The solution of synthesized silver nanoparticles was centrifuged for 30 min at 10,000 rpm for FTIR measurements.

#### 2.4.4. Scanning Electron Microscope Analysis (SEM)

To determine the structure of the obtained nanoparticles, SEM was used. The dried samples were placed on a double conductive tape fixed on a sample holder at a normal temperature. A platinum-gold coating was applied to the samples to increase conductivity. After this, the samples were visualized at 80 kV voltage.

### 2.5. In Vitro Biological Application

#### 2.5.1. Antimicrobial Activity 

All the equipment and media were autoclaved for 30 min at 115 °C and 15 psi. The disc diffusion method was performed to check the bacterial activity against Gram-positive *Bacillus cereus* and *Bacillus paramycoides* and Gram-negative *E. coli.* Stock LB broth was prepared to refresh the bacterial stain, from which 5 mL was taken in a falcon tube and injected into the pure culture of *E. coli, Bacillus cereus,* and *Bacillus paramycoides*, which was dipped into the nutrient broth incubated in a shaking incubator round the clock at 37 °C. A stock solution of Mueller Hinton Agar (MHA) was prepared for Petri dishes, 20 mL solution was put into each Petri dish and left until it solidified, and 1 mL of the overnight grown culture of each stain (*E. coli*., *Bacillus cereus,* and *Bacillus paramycoides*) was spread on the Mueller Hinton Agar plates and placed on an antibiotic disc (amoxicillin and cefpodoxime). 5 μL (4 mg/mL in DMSO) 25 mM, 1 M of AgNPs, and 1 mL of pure plant extract were placed on a petri dish and incubated for 24 h at 37 °C. The inhabitation zone around the disk was calculated and compared to the positive control. The antibacterial activities of the respective solutions were confirmed by the appearance of an inhibition zone around the disc, which was also previously recorded by Kivrak et al. [28]. If the zone of inhibition is equal to or greater than 12 mm, then it is significant [29]. 

#### 2.5.2. Cell Culture for Cell Line 

Cryopreserved cancer cells (HeLa and MCF-7) were thawed, centrifuged, re-suspended, transferred to flasks for culturing, and incubated overnight at 37 °C in a standard CO_2_ incubator. The HeLa lines were cultured in RPMI-1640 medium containing 10% FBS and 1% penicillin/streptomycin. MCF-7 cells, however, were cultured in DMEM containing 1% penicillin/streptomycin and 15% FBS. The cells were detached using 1 mL of trypsin-EDTA, diluted 10×, after three washes with sterile PBS. Cells were centrifuged for 3 min at 1500 rpm after being given 5 mL of media. The supernatant was removed, and the cells were suspended again, counted, and diluted as required.

#### 2.5.3. Assay for Cell Viability

MTT-based cell viability assessment was carried out to investigate the cytotoxic potential of silver nanoparticles in adenocarcinoma cells of the human breast, MCF-7, and adenocarcinoma cells of the human cervix, HeLa. Flat-bottomed, 96-well culture plates were used to culture (2.5 × 10^4^/mL) the cells in a volume of 90 µL and were held in a 5 percent CO_2_ incubator at 37 °C. AgNPs-treatment of the cells was performed (1 mg/mL) after an overnight incubation, followed by a further 24 h incubation. To crystallize the viable cells, the media were expelled, and MTT reagent (100 µL) was applied to all wells. The culture plate was incubated at 37 °C for 4 h with 5% CO_2_ before 100 µL of the reagent was added (1:1 of 10% SDS and 50% isopropanol). It was then incubated at room temperature for another 30 min, and a microplate reader at a 570 nm wavelength (Bio-Tek ELx 800TM, Winooski, VT, USA) measured the optical density. The findings were determined as % inhibition scores with the average of three different values (SEM) from all the experiments, which were done in triplicate. GraphPad Prism 5.0 Software Inc., San Diego, CA, USA, was used to calculate the inhibitory concentration (IC_50_) values for derivatives that showed equal to or greater than 50% inhibition.

### 2.6. Physicochemical Parameters

To explore the strength and nature of the interaction between AgNPs and solvents, thermodynamic parameters, such as density, specific heat, and thermal conductivity with water and without water, were derived to explain the physicochemical behavior after mixing the AgNPs. Density, specific heat, and thermal conductivity were measured using a mathematical equation at various temperature values in the range of 15–45 °C for pure silver nanoparticles (AgNPs) and nanoparticles mixed with water. Furthermore, at the same temperature ranges, the data were taken by mixing the AgNPs with water in different ratios—1:2, 1:4, and 1:6 (in mL)—as shown in Table 1, Table 2 and Table 3.

### 2.7. Mathematical Formulation

The momentum equation with coupled mixed convection and a uniform magnetic field:

(1) $$u\;\frac{\partial u}{\partial x}+v\;\frac{\partial u}{\partial y}=\frac{K}{\rho_{nf}\delta }\frac{\partial }{\partial y}\left({\left|\frac{\partial u}{\partial y}\right|}^{n-1}\frac{\partial u}{\partial y}\right)+\frac{1}{\rho_{nf}}\lambda T-\frac{\sigma B_{0}{}^{2}U_{0}}{\rho_{nf}}u-\frac{\nu_{nf}}{\rho_{nf}K^{*}}u-\frac{F}{\rho_{nf}}u^{2}$$

Heat transfer equation:

(2) $$u\;\frac{\partial T}{\partial x}+v\;\frac{\partial T}{\partial y}=\frac{\kappa_{nf}}{{\left(\rho Cp\right)}_{nf}}\frac{\partial }{\partial y}\left({\left|\frac{\partial T}{\partial y}\right|}^{n-1}\frac{\partial T}{\partial y}\right)+\frac{\kappa_{nf}}{{\left(\rho Cp\right)}_{nf}}{\left(\frac{\partial u}{\partial y}\right)}^{2}$$

Boundary conditions are:

(3) $$\begin{array}{l} u\;=\;0,v=0,T=0\;at\;y=0\\ u\;={\;U}_{0},v=0,T=T_{0}\;at\;y\to \infty \end{array}$$

where $U_{0}$ velocity of the sheet at *x* = 0, $T_{0}$ temperature at the sheet surface, $\rho_{nf}$ density of nanofluid, $\mu_{nf}$ viscosity of nanofluid, ${\left(\rho Cp\right)}_{nf}$ specific heat of the nanofluid.

Nanofluids are:

(4) $$\begin{array}{l} \frac{\mu_{nf}}{\mu_{f}}=\frac{1}{{\left(1-\varphi \right)}^{2.5}},\frac{\rho_{nf}}{\rho_{f}}=1-\varphi +\varphi \frac{\rho_{s}}{\rho_{f}},\frac{\kappa_{nf}}{\kappa_{f}}=\frac{1+\kappa_{f}-2\varphi (\kappa_{f}+\kappa_{s})}{1+\kappa_{f}+\varphi (\kappa_{f}+\kappa_{s})}\\ \frac{{\left(\rho Cp\right)}_{nf}}{{\left(\rho Cp\right)}_{f}}=1-\varphi +\varphi \frac{{\left(\rho Cp\right)}_{s}}{{\left(\rho Cp\right)}_{f}} \end{array}$$

Similarity transmissions are as follows:

(5) $$\psi =X^{\frac{1}{n+1}}f\left(\eta \right),T=X^{-1}\theta \left(\eta \right),\eta =\frac{Y}{X^{\frac{1}{n+1}}}$$

(6) $$\mathrm{And}\;u=\frac{\partial \psi }{\partial x},v=-\frac{\partial \psi }{\partial x}$$

The following similarity transformations [30] are considered for the reducing PDE’s to ODE’s system,

(7) $$f^{\prime \prime \prime }=\frac{{\left|f^{\prime \prime }\right|}^{1-n}}{n}\frac{\mu_{nf}}{\mu_{f}}\left(Mf^{\prime }-\lambda \theta -\frac{\rho_{f}}{\rho_{nf}}\frac{1}{n+1}ff^{\prime \prime }+K_{1}{}^{*}f^{\prime \prime }+Frf^{\prime \prime }{}^{2}\right)$$

(8) $$\theta^{\prime \prime }=-\frac{\mathrm{Pr}{\left|f^{\prime \prime }\right|}^{1-n}}{\frac{\kappa_{nf}}{\kappa_{f}}}\left(f^{\prime }\theta +\frac{1}{n+1}f\theta {}^{\prime }+\frac{n-1}{n\mathrm{Pr}}\frac{1}{\left|f^{\prime \prime }\right|}\left(Mf^{\prime }-\lambda \theta -\frac{1}{n+1}ff^{\prime }\right)+\frac{{\left(\rho Cp\right)}_{f}}{{\left(\rho Cp\right)}_{nf}}Ecf^{\prime \prime }{}^{2}\right)$$

## 3. Results

### 3.1. Color Change of Solution

**Figure 1:**
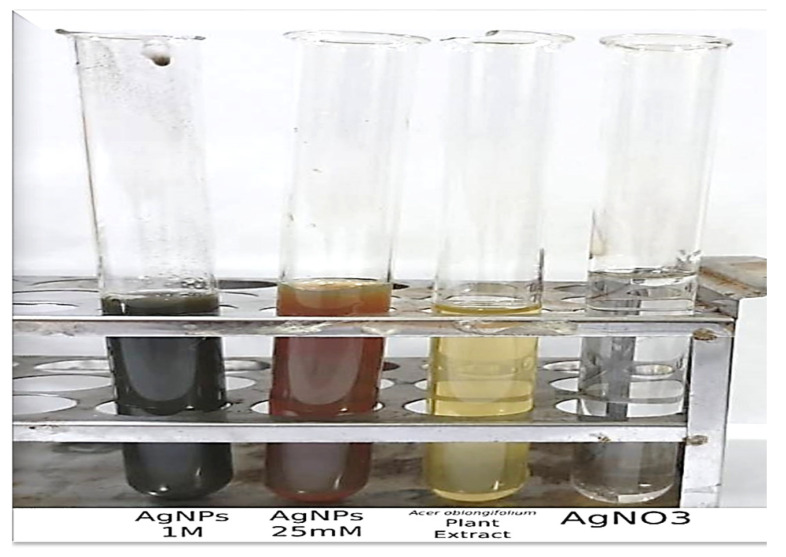
Synthesis of the silver nanoparticles indicated by color change.

The *Acer oblongifolium* plant reduced the Ag ions, and the color of the solution changed from yellow to black and brown with the addition of AgNO_3_ after 24 to 48 h at room temperature, as shown in Figure 1. The silver nanoparticles were formed using a 1 to 10 ratio (1:10) for this 90 mL of plant extract and 10 mL of silver nitrate solutions mixed. Therefore, as the incubation time passed, the color intensity increased, indicating the reduction of Ag ions and the formation of AgNPs.

### 3.2. UV Visible Spectroscopy 

**Figure 2:**
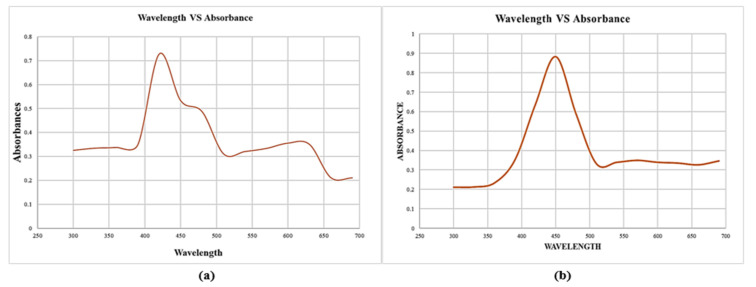
UV visible spectroscopy for silver nanoparticles synthesized using *Acer oblongifolium* plant extracts. (**a**) Synthesized from 25 mM precursor solution of AgNO_3_, (**b**) synthesized from 1 M solution of AgNO_3_.

The synthesis and stability of reduced AgNPs in colloidal solution were examined using a UV visible spectrophotometer. The maximum absorbance at 450 and 420 nm was observed in the visible UV spectra, as shown in Figure 2, which corresponds to the surface plasmon resonance (SPR) of the AgNPs. Surface Plasmon Resonance (SPR) patterns are commonly used as indicative tools for metal nanoparticle formation, as SPR depends on various parameters, such as size and medium dielectric constant [31]. The Ag^+^ ions were reduced extracellularly, indicating the formation of silver nanoparticles.

### 3.3. X-ray Diffraction (XRD) Analysis

**Figure 3:**
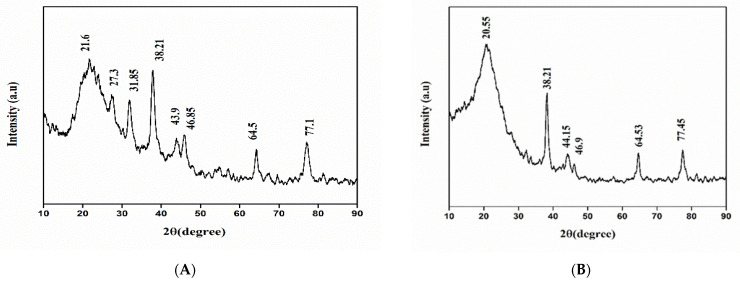
XRD pattern of silver nanoparticles synthesized using *Acer oblongifolium* extract. (**A**) Synthesized from 25 mM solution of AgNO_3_, (**B**) synthesized from 1 M solution of AgNO_3_.

Phase identification, purity, and structure of green synthesized AgNPs were determined by X-ray diffraction. Confirmation of crystalline nature and purity of AgNPs were conducted by XRD pattern with strong diffraction peaks analyzed at different 2θ, i.e., 20.55°, 38.21°, 44.15°, 46.9°, 64.53°, and 77.45° for 1M AgNPs, and for 25 mM diffraction peaks were 21.6°, 27.3°, 31.85°, 38.21°, 43.9°, 46.85°, 64.5°, and 77.1°. Diffraction peaks of AgNPs were confirmed at 38.21°, 46.9°, and 64.53° with miller indices of 111, 200, and 220, as shown in Figure 3. The average crystallized sizes of 25 mM and 1 M green synthesized AgNPs were predicted to be 3 nm and 5 nm.

### 3.4. Fourier Transform Infrared (FTIR) Spectroscopy 

**Figure 4:**
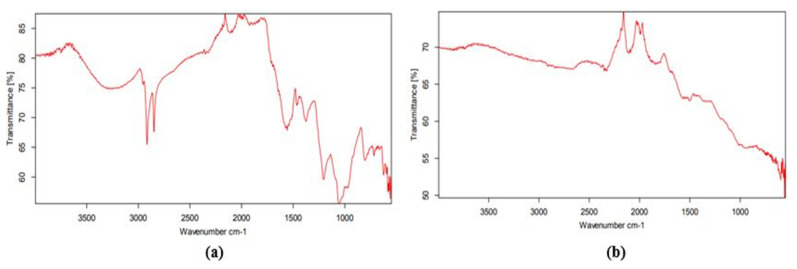
FTIR spectrum of silver nanoparticles synthesized using the leaf extract of *Acer oblongifolium* and AgNO_3_: (**a**) 25 mM precursor solution of AgNO_3_, (**b**) 1 M precursor solution of AgNO_3_.

The FTIR spectrum (Figure 4) of the reduced AgNO_3_ solution and capped by secondary metabolites with plant extracts of *Acer oblongifolium* revealed significant absorption peaks at 2865.16, 2031.92, 1478.96, and 845.35 cm^−1^. The appearance of the instinct band at 2865.16 cm^−1^ (C-H) showed alkanes present, the band at 2031.92 cm^−1^ confirmed the presence of alkynes, and the band at 1478.96 cm^−1^ revealed the N-C and N=C groups. The C-O group at 845.35 cm^−1^ depicted the presence of =CH in aromatic compounds in the biosynthesized silver nanoparticles [32], which is in accordance with previous studies [33,34,35]. 

### 3.5. Scanning Electron Microscope (SEM)

**Figure 5:**
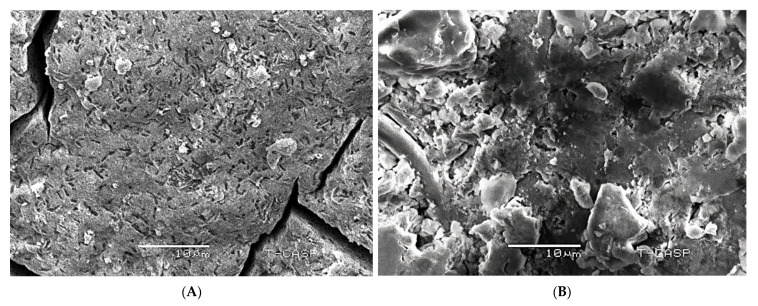
(**A**) SEM micrograph of silver nanoparticles synthesized using the 25 mM solution of AgNO_3_ and leaf extract of *Acer oblongifolium*; (**B**) SEM micrograph of silver nanoparticles synthesized using the 1 M solution of AgNO_3_ and leaf extract of *Acer oblongifolium*.

The surface morphology and particle size of the green synthesized AgNPs were determined using a scanning electron microscope (SEM). A typical scanning electron micrograph identifies that AgNPs are rod-like in shape. Moreover, the average crystalline sizes for 25 mM and 1M AgNPs were found to be 5 nm and 8 nm, respectively; this is similar to the size predicted by XRD, as shown in Figure 5.

### 3.6. In Vitro Biological Application 

#### 3.6.1. Antibacterial Activity 

**Figure 6:**
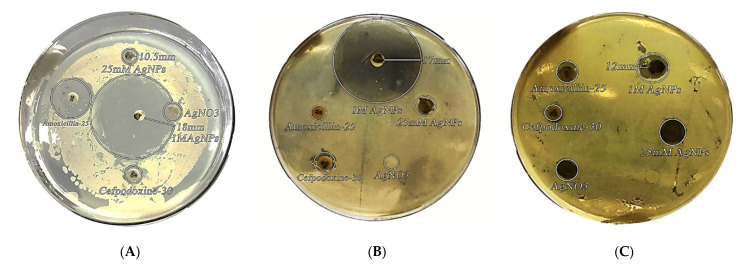
Bactericidal activity of biosynthesized AgNPs from *Acer oblongifolium* against (**A**) *E. coli*, (**B**) *B. cereus*, and (**C**) *B. paramycoides*.

The antimicrobial activity of silver nanoparticles synthesized from *Acer oblongifolium* plant extract was studied using the disc diffusion method against *Bacillus cereus*, *Bacillus paramycoides,* and *E. coli*. Figure 6 shows the diameter of zones of inhibition around each disc with silver nanoparticle solution. The antibacterial activity of AgNPs synthesized from *Acer oblongifolium* extracts was shown to be highest against *E. coli* (18 mm), lower against *Bacillus cereus* (17 mm), and lowest against *Bacillus paramycoides* (12 mm).

#### 3.6.2. Physicochemical Parameters

**Figure 7:**
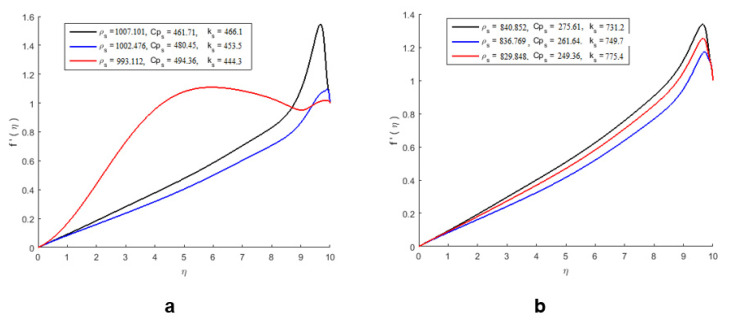
The effect of pure nanoparticle parameters on the velocity profile of pure nanoparticles and mixed nanoparticles. (**a**) Velocity profile of pure nanoparticles. (**b**) Velocity profile of nanoparticles mixed with water.

Density, specific heat, and thermal conductivity were measured using a mathematical equation at various temperature values in the range of 15–45 °C for pure silver nanoparticles (AgNPs) and nanoparticles mixed with water. Furthermore, at the same temperature ranges, the data were taken by mixing the AgNPs with water in different ratios 1:2, 1:4, and 1:6 (in mL). The physicochemical parameters, including density, specific heat, and thermal conductivity data, are given in Table 1, Table 2 and Table 3 and Figure 7a,b.

The effect of pure nanoparticles’ parameters on the velocity profile without mixing with water is shown in Figure 7a, and the effect of nanoparticles on the parameters of the velocity profile after mixing with water is shown in Figure 7b. The deplanement of the volume fraction parameters positively affected the nanofluid velocity. By improving the nanoparticles, the thickness of the boundary layer of the flow reduces, which results in an increase in the velocity of the nanofluid. However, we observed that the behavior of the velocity field is opposite in Figure 7b.

**Figure 8:**
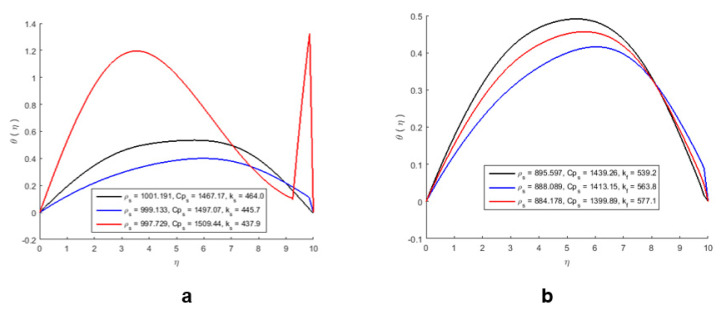
The effect of nanoparticles on the temperature profile of pure nanoparticles (**a**) and mixed nanoparticles (**b**).

The effect of nanoparticles on the temperature profile is shown in Figure 8a,b, and it can be noticed that the pure nanoparticles show more heat-absorbing capacity, while the nanoparticles mixed with water show less heat-absorbing capacity. As a result, the temperature of the nanofluid increases, which also destroys heat; therefore, the average temperature of the fluid decreases (Figure 8b).

**Figure 9:**
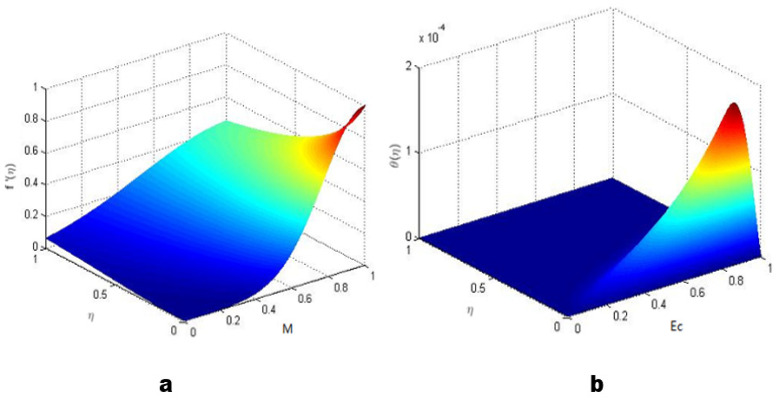
3D representation of the velocity and temperature of AgNPS: (**a**) velocity and (**b**) temperature.

The 3D representation of the velocity and temperature of the AgNPs is shown in Figure 9.

#### 3.6.3. Antiproliferative Activity against MCF-7 and HeLa Cell Lines 

IC_50_ values of AgNPs synthesized from *Acer oblongifolium* plant extract against MCF-7 and HeLa cell lines. After 48–72 h of incubation, MCF-7 showed high antiproliferative activity in comparison to the HeLa cell line, with values of 9.43 and 6.22 µg/mL, respectively.

## 4. Discussion

The color transformation to dark brown from the original yellow confirmed the formation of silver NPs from *Acer oblongifolium* plant extract. Prasad and Elumalai [36] noticed that silver nanoparticles come in a variety of colors, ranging from light yellow to brown. Furthermore, because of surface plasmon excitation fluctuations in silver nanoparticles, Prasad et al. [37] observed that these nanoparticles, in aqueous solutions, gave out a yellowish-brown color. The maximum absorbance peak for silver nanoparticles synthesized from the *Acer oblongifolium* plant extract was observed at 450 nm using the UV visible spectrum. According to Prasad and Elumalai [36], a 430–450 nm peak of absorption is observed in the silver NPs’ spectra present in the reaction media, whereas Kumar et al. [38] narrowed the gap and reported an absorbance peak of 438 nm. Moreover, unmapped peaks were also determined that indicate the phytochemical’s presence on the surface of AgNPs, which aids in capping. Moreover, the average particle sizes predicted by Image J software for 25 mM and 1 M AgNPs were 5 nm and 8 nm. This was in close proximity to the size obtained from XRD [39]. Plant extracts’ reduction by stabilizing agents plays a role in the reduction of Ag^+^ ions to Ag nanoparticles, according to FTIR study by Iqbal et al. [40]. Hemlata et al. [22] observed that silver nanoparticles bind to the carboxyl or amino groups of extracted proteins. The amine (–NH), hydroxyl (–OH), and carboxyl (–C=O) groups of leaf extracts are primarily engaged to fabricate silver nanoparticles, according to studies conducted by [41]. Scanning electron microscopy (SEM) was utilized to determine particle size as well as morphology of biosynthesized AgNPs [42]. The SEM micrograph predicted the morphology, i.e., rod-like shape. Relevant morphological studies have also been found in the literature [43]. 

*Acer oblongifolium*-derived silver nanoparticles have a potent inhibitory effect on *E. coli, Bacillus cereus*, and *Bacillus paramycoides*. Silver nanoparticles synthesized from *Acer oblongifolium* leaves are very small size, due to which they have unique physical and chemical properties. Nanoparticles have a more germicidal effect than the mass of silver metal because of the reduction of size, increase of the ratio of surface to volume of nanoparticles, and increase of the contact area with microorganisms.

Similarly, Jain et al. [44] published a human pathogen antibacterial assay using silver nanoparticles synthesized from Papaya fruit extract, which was revealed to be highly toxic against bacteria that are resistant to multiple drugs. Kumar [38] also found that AgNPs were moderately toxic to *E. coli*, *Pseudomonas* species, including *putida, aeruginosa, vulgaris,* and *B. subtilis*. The pathogens’ growth was hindered by the silver nanoparticles of the *Acer oblongifolium.* We may infer that biosynthesized AgNPs are more toxic to MCF-7 cells than to the HeLa cell line, based on the results of the antiproliferative activity study.

The activation of metallothioneins and the use of Ag^+^ chelating agents to prevent cytotoxicity support this idea. The potential of the mitochondrial membrane was depolarized by silver nanoparticles. The depolarized mitochondrial membrane potential is a key element in apoptosis signaling pathways. Finally, silver nanoparticles produced early/late apoptosis and necrosis in MCF7 and HeLa cells, with early apoptosis being the primary cause of cell death. The activity of early apoptosis was 19 percent higher than that of late apoptosis and necrosis [45]. Inducing the creation of reactive oxygen species and oxidative stress, which lead to DNA damage and apoptosis, are two possible pathways of toxicity. Toxicity in AgNP-treated cells is primarily induced by the release of Ag^+^ ions into the cytosol following AgNP uptake via endocytosis and breakdown in an acidic environment. As a result, the oxidative stress, DNA damage, and cell death observed in the presence of AgNPs are mostly attributable to silver ions in the cytosol, impairing natural metabolic and cell cycle mechanisms [46]. For MCF-7 cells, silver nanoparticles synthesized with *Artemisia vulgaris* leaf extract had an IC_50_ value close to 60 g/mL [47]. The efficacy of the aqueous leaf extract of *Cucumis sativus* against MCF-7 cells was also supported by another study [41]. *Taraxacum* *officinale* methanolic leaf extract also showed improved activity against MCF-7 cell lines. The current research is the first to compare the cell viability of *Acer oblongifolium* leaf extract-mediated biosynthesized AgNPs against two cancer cell lines. The *Acer oblongifolium* AgNPs demonstrated a difference in cytotoxicity toward different cell lines due to the increased cellular absorption and retention of NPs. Because of their small size, NPs can enter cells through endocytosis and are not subjected to efflux by P-glycoprotein [48,49]. 

## 5. Conclusions

In the current study, we have revealed the simple use of a natural, low-cost biological reducing agent and *Acer oblongifolium* leaf extracts through efficient green nanochemistry methodology, avoiding the presence of toxic solvents and waste. Silver nanoparticles made from *Acer oblongifolium* leaf extract were found to be effective against human infections. The antibacterial activity of the disc diffusion approach is well documented. Because of these applications, this technology is potentially promising for the large-scale production of nanoparticles. Prepared nanoparticles can be employed as antibacterial agents in wound healing, water purification, and medicine.

## Figures and Tables

**Table 1:** Variation of density, ρ (kg/m^3^) with change in temperature (°C) and amount of solvents.

Temperature (°C)	ρ ^1b^	ρ ^2b^	ρ^3b^	ρ ^4b^
15	1007.101	894.880	863.323	840.852
20	1002.476	886.721	856.992	936.769
25	993.112	881.204	851.638	829.848
30	986.746	875.128	846.117	825.632
35	980.174	870.998	840.264	820.987
40	975.417	863.724	834.339	815.548
45	969.105	858.301	825.114	811.716

ρ ^1b^ = density of silver nanoparticles (AgNPs); ρ ^2b^, ρ ^3b^, ρ ^4b^ = density after mixing nanoparticles and water in the ratio 1:2, 1:4, 1:6 (in mL).

**Table 2:** Variation of specific heat (J/K/mol) of nanofluid (***nf***) with temperature (°C) and amount of solvents.

Temperature (°C)	ρC ^p^	ρC ^p2^	ρC ^P3^	ρC ^p4^
15	461.71	435.62	333.15	275.61
20	480.45	424.31	321.71	261.64
25	494.36	411.51	304.14	249.36
30	507.69	396.75	292.80	231.43
35	519.14	388.64	277.90	217.87
40	527.43	371.72	264.81	199.32
45	534.77	356.70	249.33	181.56

ρC ^p^ = Speed of sound of AgNPs; ρC ^p2^, ρC ^p3^, ρC ^p4^ = Specific heat of AgNPS after mixing nanoparticles and water in 1:2, 1:4, 1:6 (in mL).

**Table 3:** Variation of thermal conductivity (Knf) with temperature (°C) and amount of solvents.

Temperature (°C)	Knf ^1^	Knf ^2^	Knf ^3^	Knf ^4^
15	466.1	537.2	652.7	731.2
20	453.5	550.4	669.2	749.7
25	444.3	562.1	688.5	775.4
30	436.0	571.6	707.1	802.8
35	430.4	592.3	727.9	822.0
40	424.7	607.5	748.4	848.3
45	419.2	624.0	769.0	874.1

Knf ^1^ = Thermal conductivity of AgNPs; Knf ^2^, Knf ^3^, Knf ^4^ = Thermal Conductivity of AgNPs after mixing nanoparticles and water in 1:2, 1:4, 1:6 (in mL).

## Data Availability

All major data generated and analyzed in this study are included in this manuscript.

## Sample Availability

Samples of the compounds are not available from the authors.
